# A sutureless technique using cyanoacrylate adhesives when creating a stoma for extremely low birth weight infants

**DOI:** 10.1186/s40064-016-1852-y

**Published:** 2016-02-27

**Authors:** Satoko Nose, Takashi Sasaki, Ryuta Saka, Kyoko Minagawa, Hiroomi Okuyama

**Affiliations:** Department of Pediatric Surgery, Hyogo College of Medicine, Nishinomiya, 663-8501 Hyogo Japan; Department of Neonatology, Hyogo College of Medicine, Nishinomiya, 663-8501 Hyogo Japan; Department of Pediatric Surgery, Osaka University Graduate School of Medicine, Suita, 565-0871 Osaka Japan

**Keywords:** Extremely low birth weight infants (ELBW infants), Intestinal perforation, Intestinal stomas, Tissue adhesive glue

## Abstract

**Purpose:**

Intestinal perforation and necrotizing enterocolitis (NEC) are neonatal intestinal emergencies that are especially common in premature infants. While prompt surgical intervention, including stoma creation, is often required, the optimal surgical treatment has been controversial because of the substantial risks related to the stoma creation and management. The use of a tissue adhesive may have some advantages over the use of sutures when creating an intestinal stoma in extremely low birth weight (ELBW) infants. The purpose of this report was to present a novel approach for creating a stoma using a tissue adhesive in ELBW infants.

**Methods:**

A total of eight ELBW infants that underwent laparotomy with the creation of intestinal stomas using cyanoacrylate adhesive at our institution between 2009 and 2014 were enrolled. The clinical parameters, including the length of the operation, intra- and postoperative complications and the outcomes were evaluated.

**Results:**

The median body weight and gestational age at birth were 630 g and 24.3 weeks, respectively. The median age at referral was 11.5 days. The median length of the procedure was 58.5 min, including the inspection and resection of the intestine. All procedures were completed without any intraoperative complications. There were no postoperative complications associated with the stoma. Two patients died of the associated septic status.

**Conclusions:**

Sutureless enterostomy using cyanoacrylate adhesive is a simple technique which has the potential to reduce the incidence of complications related to the intestinal stoma in ELBW infants.

## Background

Despite recent advances in neonatal care, idiopathic intestinal perforation and necrotizing enterocolitis (NEC) occur increasingly in extremely low birth weight (ELBW) infants with high mortality and morbidity rate. The Japanese Society of Pediatric Surgeons stated that the mortality rate of intestinal perforation ranged from 16.9 % in 2003–2007, to 18.2 % in 2008–2013, and of NEC was still over 20 %. These data show that the next step to improve overall survival among ELBW infants is to overcome intestinal perforation requiring surgical management. However, the timing and method of surgical management remain controversial because of the high mortality rates of the conditions (Blakely et al. 2005).

Emergency laparotomy and diverting enterostomy for an intestinal perforation in ELBW infants are associated with a substantial risk of complications related to the stoma because of their extreme prematurity. The key factors associated with a successful abdominal surgery in ELBW infants include a less invasive procedure and better management of a stoma. Suturing for a stoma in the intestine requires a longer operation and meticulous technique to avoid penetration of the extremely thin bowel wall in ELBW. Cyanoacrylate tissue adhesive has been used to seal wounds in accident and emergency departments, and to cover surgical wounds in the eyes and during plastic surgery, including in the pediatric population (Farion et al. 2002; Singer and Thode 2004). In the field of pediatrics, there is a randomized controlled study that cyanoacrylate tissue adhesives is as alternative to subcuticular suture for pediatric herniotomies (Ong et al. 2002). Recently cyanoacrylate adhesives are applied to circumcision for boys (Voznesensky et al. 2015), to congenital cardiac surgery as skin adhesive and bronchopleural fistula in neonates (Vida et al. 2015; Saleemi et al. 2013).

Furthermore, there have been experimental studies that have shown the advantages of suture-free intestinal application using tissue adhesive, because tissue adhesives do not impair the tissue perfusion (Weiss and Haj 2001; Kanellos et al. 2002; Faion et al. 2011). However, there have so far been no reports of cyanoacrylate tissue adhesive use for stoma creation.

We have been using cyanoacrylate adhesives as a substitute for sutures to simplify the intestinal stoma creation since 2009. This retrospective study aimed to determine whether cyanoacrylate adhesives are safe and effective in ELBW infants undergoing the creation of an intestinal stoma for a perforated intestine.

## Methods

This study was approved by the local Research Ethics Board. We performed a chart review of all cases of ELBW infants that underwent laparotomy with the creation of intestinal stomas using cyanoacrylate adhesives at the Hospital of Hyogo College of Medicine between 2009 and 2014. The following demographic data and clinical outcomes were collected: sex, gestational age at birth, body weight at birth, the underlying illness, early use of antenatal steroids and indomethacin, the length of the operation and the intra- and postoperative complications.

The surgical procedure was performed in the neonatal intensive care unit because the status of these infants frequently was life-threatening.

### Surgial work-up

All patients initially received antibiotic prophylaxis (aminobenzylpenicillin and cefotaxime). Analgosedation with fentanyl and midazolam were initially used for all mechanically ventilated ELBW infants undergoing intensive care at this institute. Vecuronium bromide was used during the procedure.

### Surgical technique

**Fig. 1:**
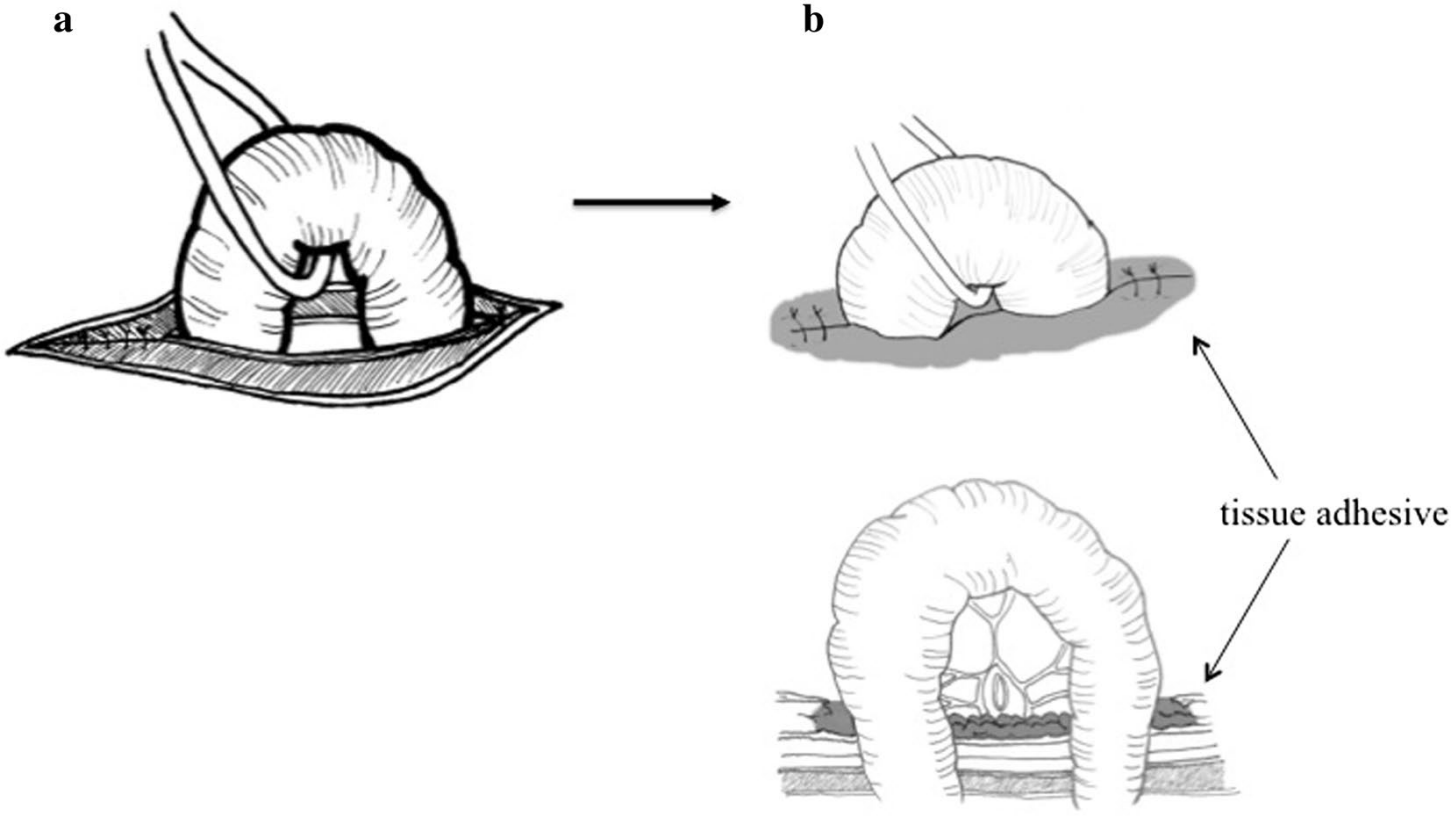
A schematic illustration of stoma creation using the cyanoacrylate adhesive. The muscle layers were closed (**a**) and the intestine was fixed to the abdominal wall using cyanoacrylate adhesive at the skin level (**b**)

The abdominal cavity was inspected through a laparotomy under local anesthesia. Thereafter, the site of intestinal perforation was identified and pulled out via the laparotomy wound. The abdominal wall was then closed in two layers. The muscle layers were closed, and the exteriorized intestine was fixed to the abdominal wall using cyanoacrylate adhesive at the end of the procedure (Fig. 1).

### Statistical analyses

The median with range was used to describe continuous variables. The statistical analysis was performed using the GraphPad Prism software package (version 6; GraphPad Software, Inc., CA, USA).

## Results

A total of 16 ELBW infants underwent laparotomy with the creation of intestinal stomas during the study period. Of those, eight infants were treated using cyanoacrylate adhesives. The patient characteristics are summarized in Table 1. The median body weight and gestational age at birth were 630 g and 24.3 weeks, respectively. The underlying illness was focal intestinal perforation in four patients, meconium-related ileus in two, meconium peritonitis in one and a congenital Treves’ field transmesenteric hernia in one infant. Three patients were small for gestational age.

**Table 1:** Patient demographics and clinical characteristics

	Median (n = 8)
Sex (M/F)	3/5
Caesarean section	8
Gestational age at birth (weeks)	24.3 (23.0–32.4)
Apgar score at 1 min	3.5 (2–7)
Apgar score at 5 min	5.5 (4–8)
Body weight at birth (g)	630 (412–926)
Early use of indomethacin	0
Early use of antenatal steroids	5
Age at operation (days)	11.5 (2–41)
Peritoneal drainage before stoma creation	0

Continuous data are expressed as the medians with the range

**Fig. 2:**
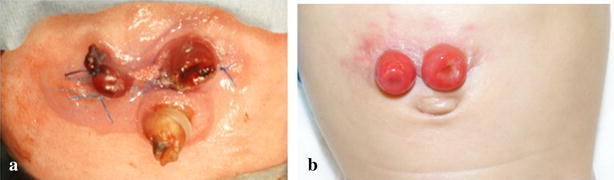
The appearance of a stoma. **a** A stoma just after creation. **b** A stoma just before closure

The median length of the operative, including inspection and resection of the intestine, was 58.5 min (range 29–106 min). None of the patients had any intraoperative complications. Although one infant required re-operation for intestinal stoma creation due to an iatrogenic injury, none had postoperative complications related to the stoma, including peritonitis caused by the migration of the stomal intestine into the peritoneal cavity, stomal prolapse or a stitch sinus. The appearance of stomas was good in most cases (Fig. 2).

The mean duration of hospitalization was 234.5 days. Two infants died of progressive liver failure; one was related to sepsis due to uncontrollable peritonitis even after the operation, the other was associated with familial idiopathic thrombocytopenia.

The follow-up time ranged from 5 months to 5 years. During the follow-up period, there were no late death or abdominal complications related to the stoma creation using sutureless technique.

## Discussion

As intestinal perforation in ELBW infants is likely to result in mortality or long-term serious consequences, and the surgical treatment of intestinal perforation in ELBW infants is still challenging (Eicher et al. 2012; Rees et al. 2007; Murthy et al. 2014). Although the standard approach for patients with an intestinal perforation is surgical resection of the involved bowel with the creation of intestinal stomas, several authors have suggested that primary peritoneal drainage under local anesthesia is better than laparotomy in ELBW infants. These studies used peritoneal drainage based on the belief that these infants could not tolerate laparotomy (Michel et al. 2004; Gollin et al. 2003). While peritoneal drainage may offer some initial advantages because it is relatively easy to perform and less invasive than surgery, a subsequent laparotomy is required for clinical deterioration and bowel trouble in 28–74 % of cases (Moss et al. 2006; Rees et al. 2008; Hunter et al. 2008; Sola et al. 2010). The special problems related to a laparotomy in ELBW infants include an increased susceptibility to infection, water and heat loss from the exposed bowel, and postoperative ventilator difficulties due to abdominal distension (Lai et al. 2010). The key factor associated with a successful abdominal surgery in ELBW infants includes the use of a minimally invasive technique. It has been reported that 2-octyl cyanoacrylate, a tissue adhesive, can be safely and effectively used as a superficial wound closure alternative (Singer and Thode 2004; Coulthard et al. 2004; Farion et al. 2002). Experimental intestinal anastomosis for the bowel using 2-octyl cyanoacrylate has been reported (Paral et al. 2011; Irkorucu et al. 2009; Kanellos et al. 2002). Cyanoacrylate adhesives allow for a shorter operation and resulted in greater satisfaction for both patients and surgeons in comparison to sutures (Farion et al. 2002). Furthermore, their use is associated with fewer infections, easier wound management, and subsequently, is more cost-effective (Wong et al. 2011; Aslam and Hunter 2009).

The complications of intestinal stomas in ELBW infants are often caused by the surgical techniques used. The thickness of the bowel wall in ELBW infants is similar to the diameter of the needles commonly used, which is 70 μm for 6-0, and 100 μm for 5-0 (Faingold et al. 2005; Epelman et al. 2007). It is therefore understandable that a needle stick can easily lead to a penetration of the bowel wall (Rygl et al. 2009; Faingold et al. 2005; Epelman et al. 2007). Tissue adhesives do not require needles, and thus eliminate the risk of breaking the bowel wall. Indeed, the present study showed no complications associated with stoma creation in our cases. The appearance of the stoma was relatively good, and it was easy to manage the skin around the stomas.

In this study, the use of cyanoacrylate adhesive did not reduce the length of the operation compared to the prior cases where sutures were used (data not shown). The length of the operation is affected not only by surgical procedures, including inspection of the affected bowel and stoma creation, but also the degree of intraperitoneal adhesion due to peritonitis. Thus, given the complicated nature of the procedure and the poor general health of ELBW infants, it was not surprising that there was no significant decrease in the length of the operation. The mortality of affected patients is associated with various factors, such as sepsis, peritonitis, the general condition and the degree of existing IVH (intraventricular hemorrhage) at the time of the surgery. This study demonstrated that the use of the liquid tissue adhesive for creating a stoma in ELBW infants could reduce the incidence of postoperative complications related to stoma creation, thus improving the outcome for the patients. Cyanoacrylate adhesives can simplify the surgical techniques, and thus make such patients easier to manage.

In conclusion, this study demonstrated that the use of cyanoacrylate adhesives in ELBW infants may have the potential to reduce the length of the operation and the incidence of complications related to the stoma. Although accumulation and analysis of large numbers of ELBW infants and cumulative data of using this sutureless technique are needed to verify this safety observation, this pilot study showed the use of cyanoacrylate tissue adhesives is recommended as alternative for stoma creation in these premature infants.
